# Concentration dependent Electrospray Ionisation Mass Spectrometry and Tandem Mass Spectrometry (MS/MS ) studies on (E,E)-1-[5-(1,3-benzodioxol-5yl)-1-oxo-2,4-pentadienyl]- piperidine (Piperine) and its analogues

**DOI:** 10.1186/2193-1801-2-427

**Published:** 2013-08-31

**Authors:** Ravi K Khajuria, Neha Sharma, Jawahar L Koul, Mahendra K Verma

**Affiliations:** Analytical Chemistry Division (Instrumentation), CSIR- Indian Institute of Integrative Medicine, Canal Road, Jammu, 180001 India; Bio-Organic Chemistry Division, CSIR- Indian Institute of Integrative Medicine, Canal Road, Jammu, 180001 India

**Keywords:** Electrospray ionisation, Preformed ions, Product ions, Piperine and its analogues, Bioavailability, *Piper nigrum*

## Abstract

Studies on piperine ((M_**1**_)) and its synthetic analogues (M_**2**–**18**_) by positive electrospray ionisation mass spectrometry were carried out in the flow injection mode of analysis in methanol. The MS experiments on these compounds at concentration 5 ng/μL or above yielded dimeric ionic species [2 M + Na]^+^ which revealed that piperine and its analogues exhibit clustering of ions when the solutions of these compounds at concentrations 5 ng/μL or above were allowed to move through the electrospray interface of the mass spectrometer. The same clustering of the ions was not observed when the solutions of the same compounds at concentrations below 5 ng/μL were used for similar studies. The formation of the clusters was further confirmed by tandem mass spectrometry (MS/MS) studies wherein the fragmentation of dimeric ionic species [2 M + Na]^+^ led to the formation of sodium adducted monomeric ionic species [M + Na]^+^. The MS measurements of these compounds by Atmospheric Pressure Chemical Ionisation (APCI) were on expected lines as there was no clustering of the ions in case of APCI-MS measurements.

## Introduction

Piperine, chemically known as (E,E)-1-[5-(1,3-benzodioxol-5yl)-1-oxo-2,4-pentadienyl]- piperidine is the major alkaloid of *Piper nigrum* plant*.* The fruit of the plant is commonly known as black pepper. The increased interest in recent years in piperine is because of its undisputed property of exhibiting a potent chemoprotective effect against procarcinogens and a host of numerous other biological activities (Reen & Rashment 1997; Koul et al. 2000; Bhardwaj et al. 2002; Khajuria et al. 2002; Koul & Kapil 1993; Cole 1985; Bajad et al. [Bibr CR1][Bibr CR2]; Timmers 1994). The molecule has attained such an important position in today’s drug discovery programme that the researchers feel tempted to develop green technologies for its isolation in pure form either by microwave (Raman & Gaikar 2002a) or by hydrotropic solubilisation (Raman & Gaikar 2002b). These new technologies for the extraction of piperine are being considerd to enable its direct use in various medicinal preparations. Black pepper has been Generally Recognised As Safe (GRAS) food additive by WHO.

In continuation of our studies on piperine (Bajad et al. [Bibr CR3][Bibr CR4]) its importance in drug discovery and the application of compound in large number of drug formulations.

Recently there have been synthesized a novel series of substituted piperine analogues in our institute. The aim of the study was to identify potential leads as bioenchancer for anti-cancer agents. There are some piperine analogues which are having appreciable bioactivity (Sachin et al. 2010; Najar et al. 2011; Sheikh et al. 2013) and can act as a bio enhancer. Keeping in mind the importance of the compounds, there is a need to understand the mass spectrometric behavior of the compounds.

ESI is undoubtedly the highly sensitive and softest ionization technique (Fenn et al. 1990) and Quadrupole Ion Trap is an extraordinary device which functions both as an ion store and mass spectrometer (Raymond 1997) of considerable mass range and variable mass resolution.

Therefore, we decided to carry out the concentration dependent Electrospray Ionisation Mass Spectrometry (ESI-MS) and tandem mass spectrometry (MS/MS) studies on this molecule (M_**1**_) and its analogues (M_**2-18**_).

## Experimental

### Materials

Piperine (HPLC purity > 99.0%) was isolated from *Piper nigrum* whereas all other analogues of piperine were synthesised. HPLC grade methanol (Rankem make) was supplied by Ranbaxy Laboratories, Mohali, India and was used without further purification. Individual stock solutions were used for infusion of each analyte into the MS system. All the solutions were passed through 0.45 μm filters before their use. For the analysis of Mixtures of piperine and its synthetic analogues (M_**2-18**_) the equiconcentration mixtures of piperine with these analogues were mixed in equal volumes for MS and MS/MS infusion experimental studies.

### Mass spectrometry

Mass spectral analyses were performed with Bruker Esquire 3000 mass spectrometer provided with ESI ion source and the ion trap as the mass analyzer.The system was equipped with an orthogonal spray ion source and Bruker Daltonics version 5.0 software (Bruker Daltonics, Germany) was used for instrument control and acquisition of the mass spectrometric data. Samples were infused into the electrospray interface using a syringe pump (Cole Parmer, 74900 series) at a flow rate of 3 μL/min. ESI- MS spectra were acquired in positive ion mode as there was no ionization in the negative mode for these compounds by scanning over a m/z range of 50–1000. Nitrogen was used both as nebulizing and desolvation gas at a flow rate of 6 L/hr. The fragmentation of the molecular ion peaks preferably called as quasi molecular ion peaks was carried out after the selection of the precursor ions in quadrupole ion trap. The ion transference efficiency from the ion source to the ion trap was automatically optimized before carrying out the MS^2^ studies. For MS^2^ experiments [2 M + Na]^+^ molecular ions were isolated in the ion trap as precursor ions and the product ion full scan spectra were recorded after fragmentation. The isolation width of m/z 0.8 was used as one of the parameter for most of the compounds to avoid the interferences of the isotopic species and most of the time the fragmentation amplitude was 2.40. Five microscans were carried out with a maximum accumulation time of 200 milliseconds & the Helium damping was introduced into the ion trap according to Manufacturer’s recommendations.

## Results and discussion

The studies were carried out under positive electrospray ionisation mass spectrometry which apparently created conditions when clustering between the ions was observed. This clustering was between the ions of same as well as the different molecules to give self as well as the cross dimeric ionic species respectively. The cross dimerisation was observed when single component at concentrations 5 ng/μL or above was injected into the electrospray interface while the cross dimeric ionic species were observed when the mixture of two components each having the concentration of 1.5-3 ng/μl or above were allowed to pass through the electrospray interface. These studies evidenced that at higher concentrations either there were preformed ions of these compounds in the solution or there was efficient ionisation which directly led to the clustering of the ions. Thus, ESI-MS of piperine and its analogues clearly exhibited predominantly dimeric ions [2 M + Na]^+^ together with [M + Na]^+^ and [M + H]^+^ ions in solutions at concentration of 5 ng/μL and above whereas at concentrations below 5 ng/μL these compounds yielded only [M + Na]^+^ and [M + H]^+^ ionic species . Therefore one can conclude that the preformed ion formation or efficient ionization does not take place in the solutions at all the concentrations. When the mixture of M_**1**_ with any of its synthetic analogue (M_**2-18**_) at concentrations more than 1.5-3 ng/μL of each were taken and mixed in equal volumes to give final concentration of 5-6 ng/μL of the given analogue and of M_**1**_ in the component mixture, formation of three sodium adducted dimeric ionic species was observed under ESI-MS. Out of these three sodium adducted dimeric ionic species one dimeric ionic species was from the M_**1**_ itself while the second was from its synthetic analogue used in the given mixture. The third dimeric ionic species was formed by the adduction of ions from one molecule each of M_**1**_ and its synthetic analogue (M_**2-18**_). First and second dimers were due to self dimerisation or clustering of the ions of the same molecule whereas the third dimer was as a result of cross dimerisation of M_**1**_ with any of the given analogue (M_**2-18**_). The results have been detailed in Table 1.

**Table 1:** **Details of molecular ion peaks and the product ions obtained during MS/MS (MS2) measurements of piperine (M**_**1**_**) and its analogues (M**_**2–18**_**)**

Compound number (n)	Structure of the compound	Molecular ions (m/z) in the ESI-MS	MS/MS spectra at m/z (2 Mn + Na)^+^
**1**		286[Mn + H]^+^, 308[ Mn + Na]^+^ and 593[2 Mn + Na]^+^	308[Mn + Na]^+^
**2**		290[Mn + H]^+^, 312[2Mn + Na]^+^ and 601[2 Mn + Na]^+^	312[Mn + Na]^+^
**3**		272[Mn + H]^+^, 294[Mn + Na]^+^ and 565 [2 Mn + Na]^+^	294[Mn+ Na]^+^
**4**		248[Mn + H]^+^, 270[Mn + Na]^+^ , 517 [2 Mn + Na]^+^	270[Mn + Na]^+^
**5**		288[Mn + H]^+^, 310[Mn + Na]^+^ , 597 [2 Mn + Na]^+^	310[Mn + Na]^+^
**6**		272[Mn + H]^+^, 294[Mn + Na]^+^ , 565 [2 Mn + Na]^+^	294[Mn + Na]^+^
**7**		300[Mn + H]^+^, 322[Mn + Na]^+^ , 621 [2 Mn + Na]^+^	322[Mn + Na]^+^
**8**		286[Mn + H]^+^,308[Mn + Na] ^+^ , 593 [2 Mn + Na]^+^	308[Mn + Na]^+^
**9**		292[Mn + H]^+^, 314[Mn + Na]^+^ , 605[2 Mn + Na]^+^	314[Mn + Na]^+^
**10**		260[Mn + H]^+^, 282[Mn + Na]^+^ , 541 [2 Mn + Na]^+^	282[Mn + Na]^+^
**11**		274[Mn + H]^+^, 296[Mn + Na]^+^ , 569 [2 Mn + Na]^+^	296[Mn + Na]^+^
**12**		292[Mn + H]^+^, 314[Mn + Na]^+^ , 605 [2 M + Na]^+^	314[Mn + Na]^+^
**13**		302[Mn + H]^+^, 324[Mn + Na]^+^ , 625 [2 Mn + Na]^+^	324[Mn + Na]^+^
**14**		278[Mn + H]^+^, 300[Mn + Na]^+^ , 577[2 Mn+ Na]^+^	300[Mn + Na]^+^
**15**		306[Mn + H]^+^ , 328[Mn + Na]^+^ , 633[2 Mn + Na]^+^	328[Mn + Na]^+^
**16**		276[Mn + H]^+^, 298[Mn + Na]^+^ , 573[2 Mn + Na]^+^	298[M + Na]^+^
**17**		272[Mn + H]^+^, 294[Mn + Na]^+^ , 565 [2 M + Na]^+^	294[Mn + Na]^+^
**18**		332[Mn + H]^+^, 354[Mn + Na]^+^, 685[2 Mn + Na]^+^	354[Mn + Na]^+^

**Figure 1:**
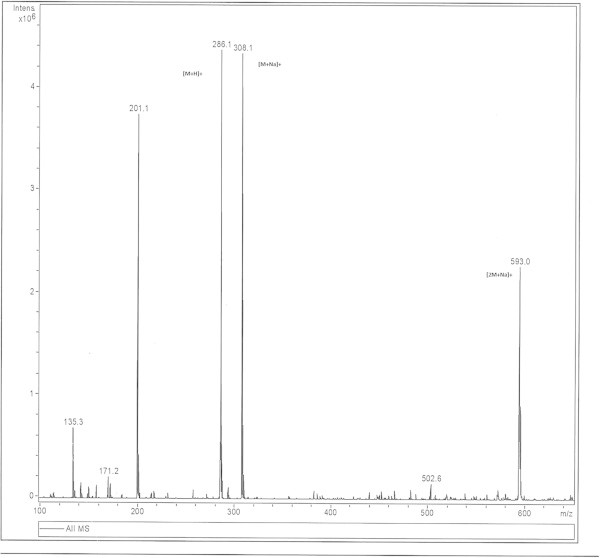
**TIC of ESI-MS of Piperine(M**_**1**_**).**

Piperine (M_1_) and all its seventeen synthetic analogues (M_2–18_) exhibited dimeric ionic species at concentrations above 5 ng/μl under the ESI interface. The dimerisation was further confirmed by MS/MS experiments (Table 2).The ESI-MS of piperine afforded the characteristic series of molecular ion at m/z 593,308 and 286 [Figure 1] which were due to [2Mn + Na]+, [M_1_ + Na] + and [M_1_ + H] + ions respectively. Low-energy collision induced dissociation (CID-MS/MS) experiments of the precursor [2 M_1_ + Na] + at m/z 593 yielded the sodiated molecule product ion at m/z 308 which was due to the formation of [M_1_ + Na] + ions. It is thus clear that the mass number at m/z 593 [2 M_1_ + Na] + is the dimer of molecular ion peak at m/z 286.

**Table 2:** **Molecular ion peaks obtained from infusion experiments of mixtures of synthetic analogues of piperine (M**_**2–18**_**) and Piperine (M**_**1**_**) and the product ions obtained during the MS/MS (MS**^**2**^**) measurements of the cross dimeric ionic peaks**

Mixture of 1 with	Molecular ion (m/z) peaks formed from the mixtures of Piperine (M_1_) with its synthetic analogues(M_2-18_)	Precursor ion (m/z) selected for MS^2^ studies	Product ions(m/z) formed from the fragmentation of the parent ions
	[2Mn + Na]^+^ n =1,2,38		
**2**	593 [2 M_1_ + Na]^+^, 601[2 M_2_+ Na]^+^ , 597[M_1_ + M_2_ + Na]^+^	597	312, 308.
**3**	593 [2 M_1_ + Na]^+^, 565[2 M _3_+ Na]^+^ , 579[M_1_ + M_3_ + Na]^+^	579	294, 308
**4**	593 [2 M _1_ + Na]^+^, 517[2 M _4_+ Na]^+^ , 555[M_1_ + M_4_ + Na]^+^	555	270, 308
**5**	593 2[M _1_ + Na]^+^, 597[2 M _5_+ Na]^+^ , 595[M_1_+ M_5_ + Na]^+^	595	310, 308
**6**	593 [2 M _1_ + Na]^+^, 565[2 M_6_+ Na]^+^ , 579[M_1_ + M_6_ + Na]^+^	579	294, 308
**7**	593 [2 M _1_ + Na]^+^, 621[2 M _7_ + Na]^+^ , 607[M_1_ + M_7_ + Na]^+^	607	322, 308
**8**	593 [2 M _1_ + Na]^+^ or [2 M_8_ + Na]^+^	593	308
**9**	593 [2 M _1_ + Na]^+^, 605[2 M _9_+ Na]^+^ , 599[M_1_ + M_9_ + Na]^+^	599	314,, 308
**10**	593 [2 M _1_ + Na]^+^, 541[2 M _10_+ Na]^+^ , 567[M_1_+ M_10_ + Na]^+^	567	282, 308
**11**	593 [2 M_1_ + Na]^+^, 569[2 M _11_+ Na]^+^ , 581[M_1_ + M_11_ + Na]^+^	581	296, 308
**12**	593 [2 M _1_ + Na]^+^, 605[2 M_12_ + Na]^+^ , 599[M_1_ + M_12_ + Na]^+^	599	314, 308
**13**	593 [2 M _1_ + Na]^+^, 625[2 M _13_+ Na]^+^ , 609[M_1_ + M _13_ + Na]^+^	609	324, 308
**14**	593 [2 M_1_ + Na]^+^, 577[2 M_14_+ Na]^+^ , 585[M_1_ + M_14_ + Na]^+^	585	300, 308
**15**	593 [2 M_1_ + Na]^+^, 633[2 M_15_+ Na]^+^ , 613[M_1_+ M_15_ + Na]^+^	613	328, 308
**16**	593 [2 M_1_ + Na]^+^, 573[2 M_16_+ Na]^+^ , 583[M_1_ + M_16_ + Na]^+^	583	298, 308
**17**	593 [2 M_1_ + Na]^+^, 565[2 M_17_+ Na]^+^ , 579[M_1_+ M_17_ + Na]^+^	579	294, 308
**18**	593 [2 M _1_ + Na]^+^, 685[2 M _18_+ Na]^+^ , 639[M_1_ + M_18_ + Na]^+^	639	354, 308

**Figure 2:**
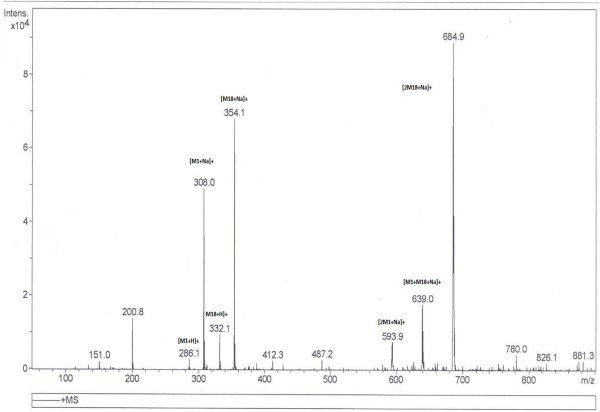
**TIC of ESI-MS of Mixture of M**_**1**_**and M**_**18**_**.**

**Figure 3:**
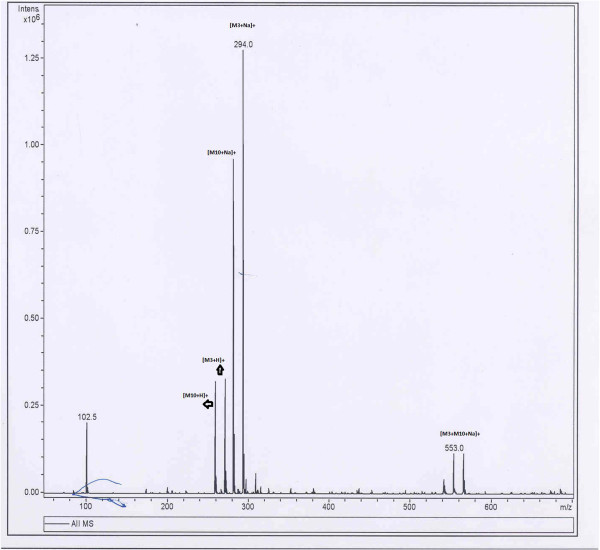
**TIC of ESI-MS of Mixture of M**_**3**_**and M**_**10**_**.**

**Figure 4:**
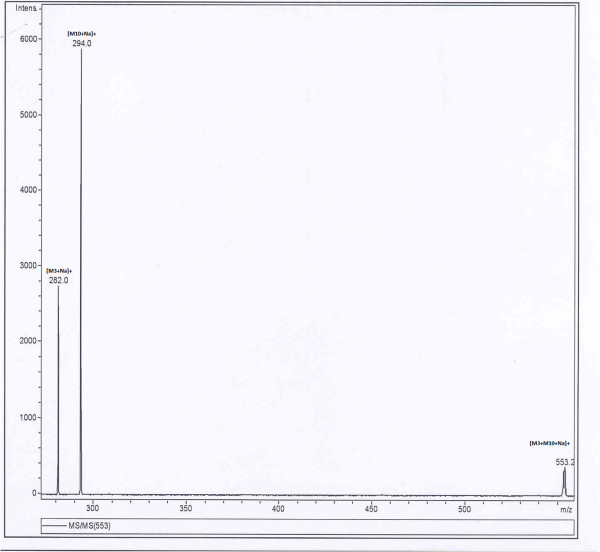
**TIC of ESI-MS/MS of Mixture of M**_**3**_**and M**_**10**_**.**

The mass number at m/z 593 which is the dimer of mass number 286 [M_**1**_ + H]^+^ has the adduction of sodium ions and mass number at m/z 308 [M_**1**_ + Na]^+^ is the monomer of piperine which also has the adduction of sodium ions. In other experiments 17 different mixtures (solutions) of piperine and its analogues at concentrations more than 2-3 ng/μl each in the mixtures were prepared. These mixtures under ESI-MS formed three sodium adducted dimeric species. The two dimeric ionic species were due to the self dimerisations of M_**1**_ and any of its given analogues and the third dimeric ionic species was due to the cross dimerisation of M_1_ with the analogue. Both in self as well as cross dimerisations, the adduction of sodium was there. For example, a mixture of solution of piperine (M_1_) and compound M_**18**_ when analysed under electrospray, three sodium adducted dimeric ionic species were observed at m/z 685, 639 and 593 [Figure 2]. Out of the three species, two exhibited molecular ion peaks at m/z 593 [2 M_**1**_ + Na]^+^ and m/z 685 [2 M _**18**_ + Na]^+^ which were as a result of self dimerisation of piperine and compound M_**18**_ ions, respectively while the third dimeric ionic species at m/z 639 [M_**1**_ + M_**18**_ + Na]^+^ was due to the cross dimerisation between the ions of M_**1**_ and those of the compound M _**18**_. The MS/MS studies of cross dimeric ionic species at m/z 639 [M_**1**_ + M_**18**_ + Na]^+^ yielded product ions at m/z 308 [M_**1**_ + Na]^+^ and 354 [M_**18**_ + Na]^+^. These product ions were due to the monomeric ionic species from M_1_ and compound M_**18**_ clearly evidencing the cross dimerisation between two different ionic species. In another example equiconcentration mixture of compound number M_**3**_ and M_**10**_ on infusion under ESI led to the formation of three dimeric ionic species. These ionic species were observed at m/z 540.9, 553.0 and 565.0 for [2 M_**10**_ + Na]^+^, [M_**3**_ + M_**10**_ + Na]^+^ and [2 M_**3**_ + Na]^+^ respectively [Figure 3]. The dimeric ionic species at m/z 540.9 and 565 for [2 M_**10**_ + Na]^+^ and [2 M _**3**_ + Na]^+^ were due to self dimerisation of compound M_**3**_ and M_**10**_ respectively whereas molecular ion peak at m/z 553 was due to cross dimerisation between compound number M_**10**_ and M_**3**_ with the adduction of the sodium ions. MS/MS of the dimeric ionic species at m/z 553 led to the formation of two product ions at m/z 282 [M_**3**_ + Na]^+^ and 294 [M_**10**_ + Na]^+^. These ionic species were the monomeric ionic species of compound M_**10**_ and M_**3**_, respectively [Figure 4].

Musharraf et al. (2010) have reported the analysis and development of structure-fragmentation relationships in withanolides using an ESI-QqTOF-MS instruments where they observed the presence of the protonated molecules [M + H]^+^ in the case of ten withanolides (steroidal lectones). Development of a validated UPLC-qTOF-MS method for the determination of curcuminoids and their pharmacokinetic study in mice has also been described by Verma et al. (2013). It has been revealed that curcumin shows the presence of [M + Na]^+^ while as other curcuminoids viz. desmethoxycurcumin and bisdesmethoxycurcumin have shown the presence of [M + H]^+^ ion. There is no dimer formation have been occurred in the above both the cases.

## Conclusions

It was observed by ESI-MS measurements that at total concentrations equal to or above 5 ng/μl of M_**1**_ and its analogues (M_**2**–**18**_) yielded sodium adducted dimeric ionic species. Under ESI-MS/MS measurements the dimeric ionic species which have the adduction of the sodium underwent fragmentation to yield sodium adducted monomeric ionic species as product ions. When the similar studies were carried out at concentrations below 5 ng/μL, M_**1**_ and each of its analogues, M_**2**–**18**_ yielded only monomeric species individually as well as when the solutions of these compounds were infused in the interface in combination. No clustering was observed between the same or different molecules. It means that the chances of clustering of the ions at higher concentrations in the gaseous state via non covalent interactions cannot be ruled out.
